# Elastic modulus and toughness of orb spider glycoprotein glue

**DOI:** 10.1371/journal.pone.0196972

**Published:** 2018-05-30

**Authors:** Brent D. Opell, Mary E. Clouse, Sheree F. Andrews

**Affiliations:** Department of Biological Sciences, Virginia Tech, Blacksburg, Virginia, United States of America; Chang Gung University, TAIWAN

## Abstract

An orb web’s prey capture thread features tiny glue droplets, each formed of an adhesive glycoprotein core surrounded by an aqueous layer. Small molecules in the aqueous layer confer droplet hygroscopicity and maintain glycoprotein viscoelasticity, causing droplet volume and glycoprotein performance to track changes in environmental humidity. Droplet extension combines with that of a thread’s supporting flagelliform fibers to sum the adhesive forces of multiple droplets, creating an effective adhesive system. We combined measurements of the force on an extending droplet, as gauged by the deflection of its support line, with measurements of glycoprotein volume and droplet extension to determine the Young’s modulus (*E*) and toughness of three species’ glycoproteins. We did this at five relative humidities between 20–90% to assess the effect of humidity on these properties. When droplets of a thread span extend, their extensions are constrained and their glycoprotein filaments remain covered by aqueous material. This was also the case during the first extension phase of the individual droplets that we examined. However, as extension progressed, the aqueous layer was progresses disrupted, exposing the glycoprotein. During the first extension phase *E* ranged from 0.00003 GPa, a value similar to that of fibronectin, a glycoprotein that anchors cells in the extracellular matrix, to 0.00292 GPa, a value similar to that of resilin in insect ligaments. Second phase *E* increased 4.7–19.4-fold. When compared at the same humidity the *E* of each species’ glycoprotein was less than 5% of the value reported for its flagelliform fibers. This difference may facilitate the coordinated extension of these two capture thread components that is responsible for summing the thread’s adhesive forces.

## Introduction

**Fig 1:**
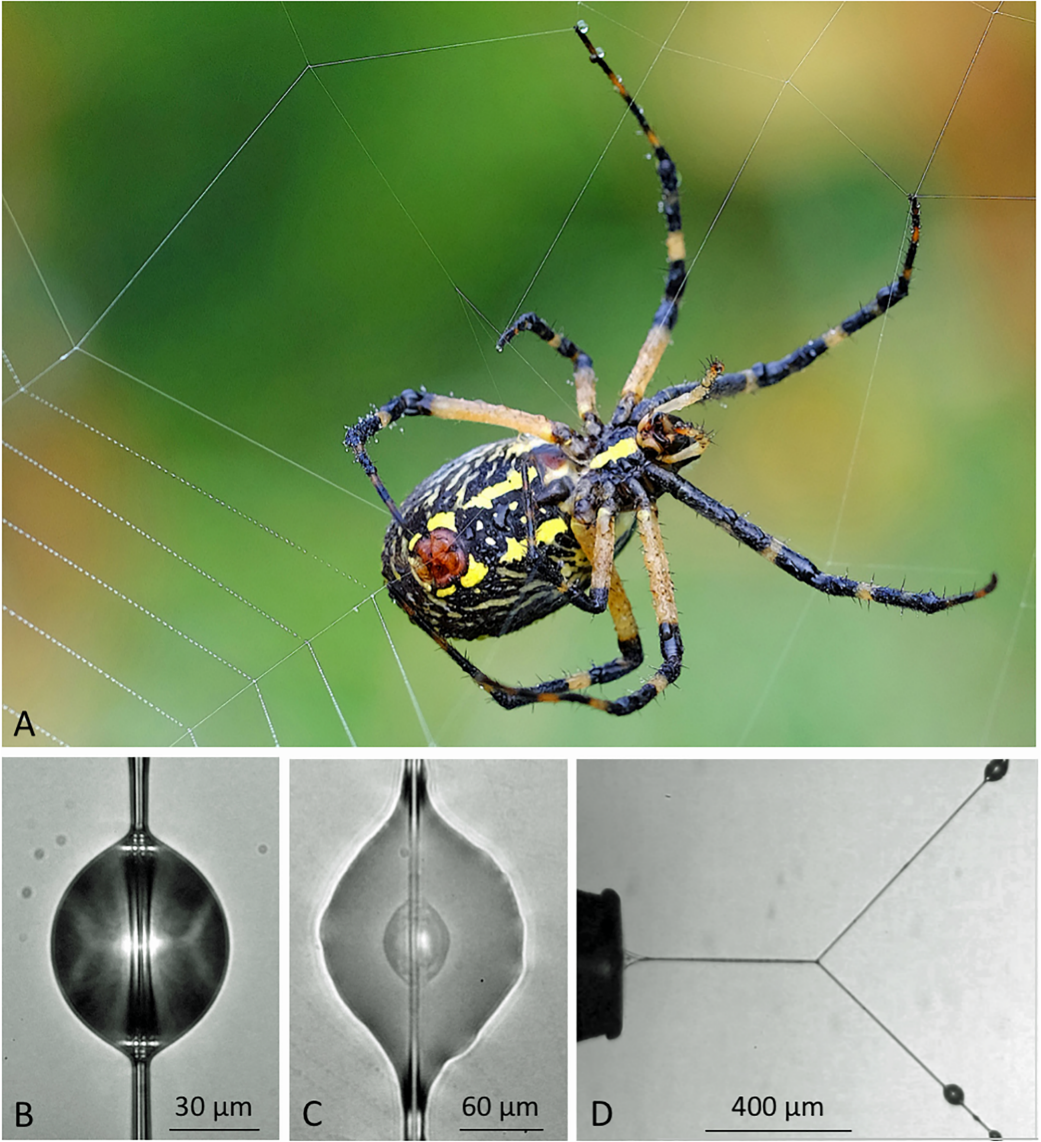
Viscous prey capture threads and their droplets. A. *Argiope aurantia* female depositing a capture thread. B. A suspended *A*. *aurantia* viscous droplet, showing paired flagelliform axial fibers, as magnified by the droplet’s curved surface, C. A flattened *N*. *crucifera* droplet showing a glycoprotein core attached to axial fibers and surrounded by aqueous material. D. An extended *N*. *crucifera* droplet, which has deflected its support line.

The multiple uses that spiders make of their proteinaceous silk threads have made an important contribution to the success of this 47,055-species clade [1–6]. Nowhere is this more evident than in the intricate webs constructed by orb weaving spiders of the superfamily Araneoidea (Fig 1A). Along with their descendants that construct webs of divergent architectures, members of this clade comprise 26% of all living spider species [7–9]. These orb webs are the products of four different silk glands, each opening at the tip of a spigot on one of the spider’s six spinnerets [10]. Attached by pyriform gland disks [11], threads from the major ampullate glands form the web’s strong, but stiff frame and radial lines that absorb and dissipate the kinetic energy of prey strikes [12–14]. Adhesive viscous prey capture threads are laid as a spiral on the web’s radii and prevent insects from escaping the web before a spider can locate, run to, and subdue them [15, 16]. These composite threads are produced by two types of silk glands that open on adjacent spigots on a spider’s paired posterior lateral spinnerets [17]. On each spinneret, two aggregate gland spigots flank a flagelliform gland spigot. As a flagelliform fiber emerges, it is coated with aqueous aggregate gland material. Threads from the two spinnerets merge and Plateau Rayleigh instability quickly reconfigures the cylinder of aggregate material that surrounds the two flagelliform fibers into a series of regularly spaced droplets, a core of adhesive glycoprotein coalescing inside of each droplet Fig 1A–1C) [18–20].

**Fig 2:**
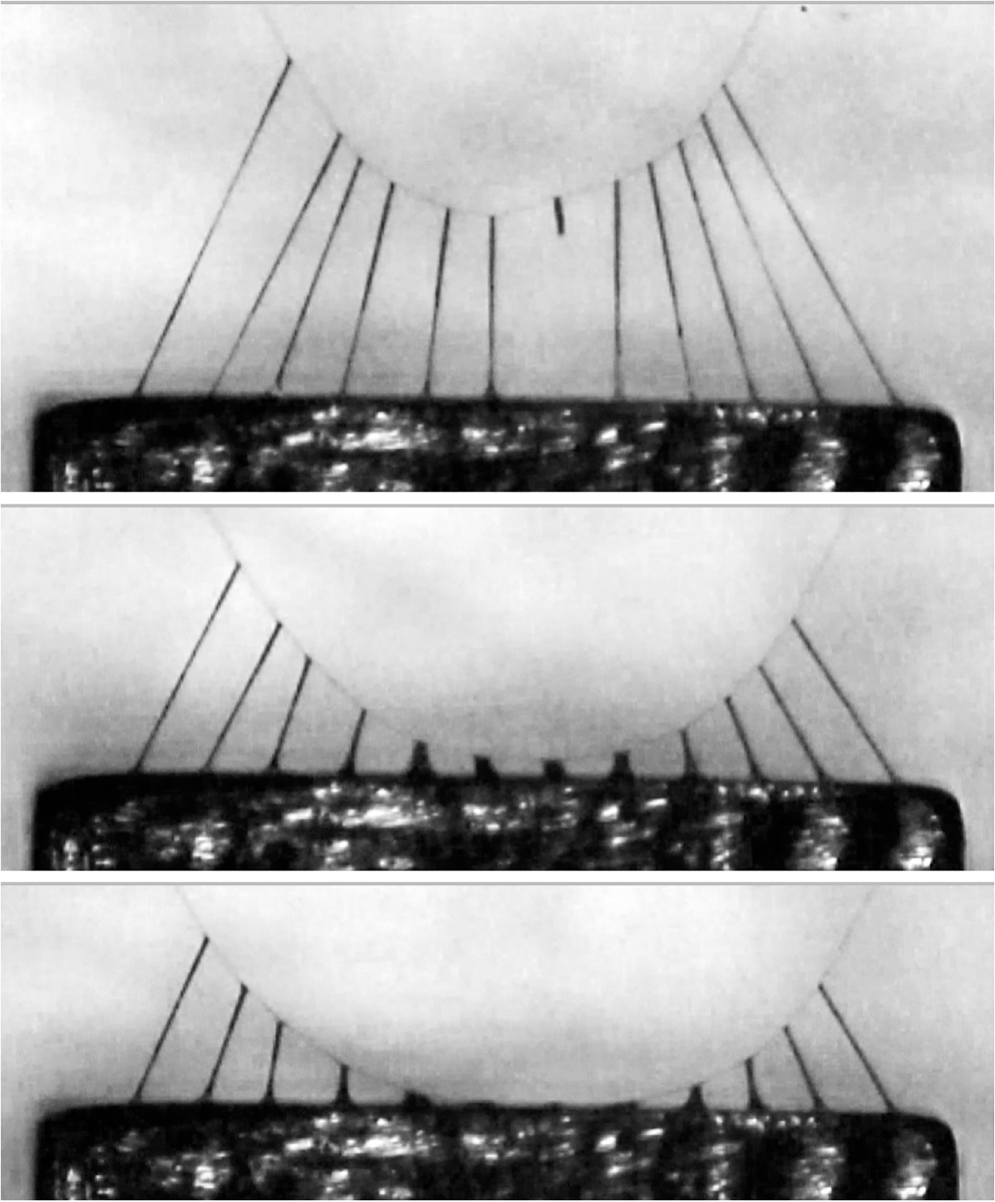
Video screen captures of a *V*. *arenata* capture thread being progressively pulled (bottom to top) from a 2mm wide contact plate. Force from the thread’s extending droplets is summed by its deflected axial line. In the top frame a droplet near the strand’s center has released from the plate, introducing an instability that will initiate adhesive failure. Thread release is more typically initiated when peripheral droplets release. Middle and top figures from Opell et al., 2018.

Unlike the anchoring adhesives of mussels and barnacles, which are enzymatically toughened to resist crack propagation that leads to failure [21–24], orb spider glycoprotein glue has evolved as a compliant, extensible adhesive (Fig 1D), a feature that is critical for a thread’s adhesion. As a struggling insect pulls on an attached capture thread, individual droplets extend, transferring force to the axial line, causing it to bow and assume a parabolic configuration (Fig 2). Thus, in suspension bridge fashion, the adhesion generated by multiple droplets is summed over a thread span [25, 26]. Hygroscopic low molecular mass compounds (LMMCs) and inorganic salts in the aqueous layer that surrounds a thread’s axial fibers and glycoprotein core facilitate thread adhesion in two ways. These compounds attract atmospheric moisture [27–30], which ensures the extensibility of both glycoprotein and axial fibers [31–34]. LMMCs also maintain the glycoprotein’s structure and enhance its surface interactions [35]. Under light microscopy proteins are visible only in the droplet’s core (Fig 1C). However, along with LMMCs, proteins are also present in the droplet’s aqueous layer [35].

Major ampullate threads, flagelliform axial fibers, and glycoprotein are all spidroins, members of the spider fibroin gene family of scleropoteins [4, 36–41]. Since orb weavers first spun viscous threads in the early cretaceous [42] the material properties and these threads and of major ampullate threads appear to have evolved in consort, tuning each to their role in prey capture. Consistent with their role of absorbing the force of prey impact, major ampullate threads are stiffer than flagelliform fibers, which support viscous capture threads [12]. Stiffness, more formally termed elastic modulus or Young’s modulus (*E*), describes a material’s resistance to being deformed elastically. Major ampullate threads have *E* values of 3.4–11.5 GPa and flagelliform fibers values of 0.012–0.08 GPa [12]. We hypothesize that to effectively implement a suspension bridge mechanism of adhesive recruitment glycoprotein *E* should be less than that of flagelliform fibers, whose values differ among species [12]. If glycoprotein is too stiff relative to a thread’s flagelliform fibers then the outer droplets of a contacting strand will release before inner droplets have extended and contributed their adhesion. To test this hypothesis we constructed stress-strain curves for three species’ viscous droplets and used these curves to determine *E* of the droplet’s glycoprotein cores. These curves also permitted us to determine the glycoprotein core’s toughness, the work required to extend the material to rupture, or, in the case of viscous threads droplets, pull-off from a contacted surface. This is an important index because droplet and axial line extension dissipate the energy of an insect’s struggles to escape from a web [43].

Under the high humidity of early morning and evening, when most orb webs are constructed, hygroscopic compounds in a viscous thread aggregate material attracts atmospheric moisture, which contributes to the volume and instability of the aggregate cylinder that initially covers the axial fibers. After droplets and their glycoprotein cores form, these compounds remain in the aqueous layer, causing droplet volume and glycoprotein volume and viscosity to track changes in environmental humidity [17, 32, 44]. For species that occupy humid stream-side or forest habitats little hygroscopicity is necessary to achieve a balance between glycoprotein adhesion and cohesion that optimizes thread adhesion [44]. In fact, hygroscopicity that is excessive reduces viscosity and leads to cohesive failure [32, 34]. However, for species that live in exposed habitats where humidity drops during the day, greater thread hygroscopicity is necessary to maintain glycoprotein viscosity. When the adhesion of five species’ capture threads were measured at different humidities, each registered the greatest value at its typical foraging humidity, but at these optimal humidities all species had similar glycoprotein viscosities [44]. Thus, natural selection has tuned the viscosity of an orb spider’s glycoprotein glue to the humidity of its habitat by adjusting the composition and concentration of LMMCs and salts that determine the aqueous layer’ hygroscopicity. To complete this picture, we characterized the *E* and toughness of glycoproteins from three of the five species included in this previous study at five humidities ranging from 20–90% relative humidity (RH). Although viscosity and elastic modulus are both affected by glycoprotein cohesion, they are not interchangeable indices. Viscosity was measured from the spreading velocity of glycoprotein on a glass surface while it remained within its native aqueous layer [44], whereas we determined Young’s modulus by extending droplets to pull-off. As physical and covalent bonds appear responsible for glycoprotein cohesion [33, 34] glycoprotein flow is unlikely to disrupt these bonds; whereas extension affords an opportunity for glycoprotein molecules to unfold and for sacrificial bonds to break [45].

The *E* and toughness of a material is determined from its stress-strain curve, which requires simultaneously measuring the force on the material and its elongation as it is extended to the point of failure. However, the small size of a viscous droplet’s glycoprotein core (Fig 1C; 821–8,625 μm^3^ at 55% relative humidity for the three species that were studied) makes it difficult to directly measure the force on single extending glycoprotein (Fig 1D). Moreover, the distribution of forces across the extending droplets of a capture thread strand is complicated [26], making it difficult to determine the force on a single droplet from the force on the entire strand. We met this challenge by using the deflection of a thread’s axial line to gauge the force on a droplet during its video-captured extension (Fig 1D).

## Methods and materials

### Species studied and spider welfare

We studied three orb weaving species: *Verrucosa arenata* (Walckenare, 1841), a diurnal species that occupies humid forests, *Neoscona crucifera* (Lucas, 1838), a nocturnal species that is found in vegetation on forest edges, where it constructs webs shortly after dusk, but continues to forage during the following day, and *Argiope aurantia* Lucas, 1833, a dirunal species found in weedy vegetation where its web experiences low humidity during the late morning and afternoon [32]. These are common and abundant species that are neither threatened nor endangered. Threads from webs constructed by 12, 14, and 14 adult females, respectively, of these species were collected from property near Blacksburg, Montgomery County, Virginia that is owned by Virginia Tech. Permission for this activity was granted by Virginia Tech’s Department of Biological Sciences, College of Science, and Office of Sponsored Programs during the review and approval of the protocols described in the research grant that supported this study. This review and the project’s approval by the United States National Science Foundation (NSF) ensured that the procedures we used complied with both Virginia Tech and NSF policies for the ethical use of animals in research studies. Only one web sample was collected from each individual spider. Spiders were not collected. Each spider either ran to or was chased unharmed to the edge of her web before a web sample was taken. The spider was, therefore, able to use the remainder of her web during the day of our collection and to construct a new web on the following day. Consequently, the impact of our thread sampling protocol on an individual spider was no greater than that of a single rainy day, which damaged her web or prevented her from spinning a new web.

### Thread collection and preparation

We collected sectors from orb-webs soon after they were constructed to ensure threads were not contaminated by dust or pollen. *Neoscona crucifera* samples were collected between 21:00 and 22:00 and *A*. *aurantia* and *V*. *arenata* between 06:00 and 08:30. We marked the position of each sampled web with flagging tape to prevent resampling. Web sectors were collected using either an 18-cm diameter aluminum ring or on a 15 x 52 cm rectangular aluminum frame. Double-sided 3M tape (3M #9086K29550360) applied to the 0.6 cm width of the ring and center bar and on the 1.2 cm wide rectangular frame secured the threads without altering the original structure and tension of the web’s threads. Immediately after collecting a web sample the ring or frame was placed in a closed container to prevent contamination. All images and videos described in the following sections were completed by 16:00 on the day web samples were collected. Procedures for characterizing droplets have been described [26, 31, 32, 46, 47] and are summarize here.

We prepared two sets of thread samples from each web, one used to determine the volume of the glycoprotein core within a droplet and one used to measure droplet extension. Threads on both samplers spanned a 4.8 mm space between supports that were covered with double-sided carbon tape (Cat #77816, Electron Microscope Sciences, Hatfield PA) to secure threads at their native tensions. On samplers used for droplet extension, we used a minuten insect pin moistened with water to slide droplets away from a focal droplet located at the strand’s center.

### Establishing test conditions

We photographed and extended droplets inside a glass covered chamber where temperature was maintained at 23° C. We monitored humidity with a Fisher Scientific^®^ Instant Digital Hygrometer and established 20% and 37% RH’s by placing a small Petri dishes with silica gel beads inside the chamber and 55%, 72% and 90% RH’s by substituting a distilled water saturated Kimwipe^®^. We made small increases in RH by forcing air through a glass cylinder packed with distilled water saturated Upsorb sheets (Diversified Biotech) into the chamber and small increases in RH by drawing room air (~ 50% RH) into the chamber.

### Determining droplet and glycoprotein volume

At each humidity, we photographed three suspended droplets and then flattened them by dropping a glass coverslip onto them from a release mechanism contained within the observation chamber, revealing each droplet’s glycoprotein core (Fig 1C). Using ImageJ [48] we measured the lengths (DL; dimension parallel to the axial fiber) and widths (DW) of three suspended droplets and the surface area of each flattened droplet and its glycoprotein core. We computed suspended droplet volume (DV using the following formula [49, 50].

$$DV=(2\pi \;*\;{DW}^{2}\;*\;DL)/15$$ (1)

We divided by a droplet’s surface area by its flattened surface area (GA) to determine its thickness, which was multiplied by glycoprotein surface area to determine glycoprotein volume (GV). For each individual and each test humidity, the mean glycoprotein-volume-to-droplet-volume ratio of three droplets was multiplied by volume this individual’s extended droplet(s) (one droplet for *A*. *aurantia* and *N*. *crucifera* and two droplets for *V*. *arenata*) to infer the volume of this droplet’s glycoprotein core.

*Neoscona crucifera* droplets did not flatten or adhere at 20% RH and, therefore, no values are available for this humidity. At 20% RH the glycoprotein cores of *V*. *arenata* did not flatten but appeared as spheres. Although cores flatten progressively as humidity increased, they continued to remained elevated above the droplets flattened aqueous layer. Therefore, we computed V. arenata glycoprotein volume as described below.

1. We determined the radius (R) of the glycoprotein core from its measured surface area (GA) as if this were an image of a bisected sphere using the formula:
  $$R=\sqrt{GA}/\pi$$ (2)
2. We determined the glycoprotein core’s spherical volume (GSV) of the glycoprotein core using the formula:
  $$GSV=\frac{4}{3}\pi {\;R}^{3}$$ (3)
3. We next used the following formula to compute an adjusted glycoprotein volume (AGV) that accounted for progressive glycoprotein volume as humidity increased.
  AGV=GSV*(GA/GAat20%RH) (4)

S1 Fig compares *V*. *arenata* AGV and GV to those of the other two species’ GV’s, confirming that AGV proved a more appropriate index for this species, which increased rather than decreased with rising humidity. S2 Fig confirms that in all three species the thickness of flattened droplets decreases as humidity increases.

### Extending droplets

**Fig 3:**
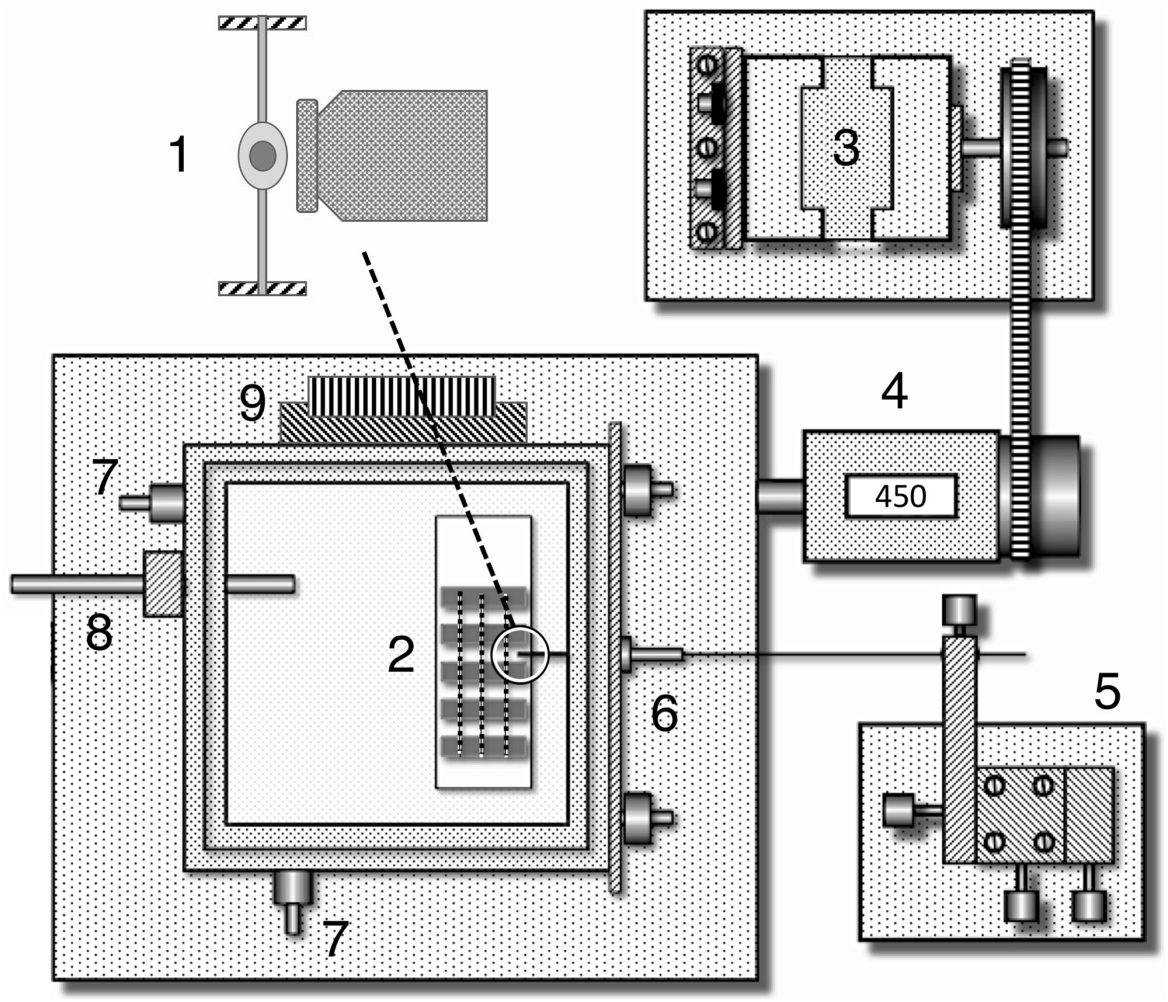
Components of the droplet extension system. 1. An isolated droplet suspended between supports with the probe’s tip positioned and ready to contact droplet. 2. Microscope slide sampler within the glass-covered humidity and temperature controlled temperature chamber resting on the microscope’s mechanical stage. 3. Stepping motor used to activate the mechanical stage’s X manipulator, which pushes the stage to the left of the image. 5. Stationary device that holds the probe stationary as the stage is moved. 6. Adjustable plate with a port for the probe to pass through, enabling the probe tip to be aligned with a thread droplet before the probe is stabilized. 7. Intake and outlet ports used to draw dehumidified air or room air into the observation chamber. 8. Hygrometer probe used to monitor chamber humidity and thermistor probe for controlling chamber humidity. 8. Peltier heating and cooling block, which maintains chamber temperature. Modified after Opell, Karinshak, and Sigler, 2011.

The droplet observation chamber rested on the stage of a Mitutoyo FS60 inspection microscope (Mitutoyo America Corp., Aurora, IL, USA) (Fig 3). We photographed each droplet prior to extension. After cleaned the 413 μm wide polished steel tip of the probe with 100% ethanol on a Kimwipe^®^, the probe was inserted through a port in the side of the chamber, aligned with a thread droplet and anchored so that it remained stable. The microscope’s stage was then advanced 500 μm, pressing the droplet against the probe tip and ensuring adhesion. A stepping motor attached to the microscope’s mechanical stage then withdrew the thread at a velocity of 69.6 μm s^-1^ while a 60-fps video recorded the droplet’s extension.

### Computing glycoprotein Young’s modulus and toughness

**Fig 4:**
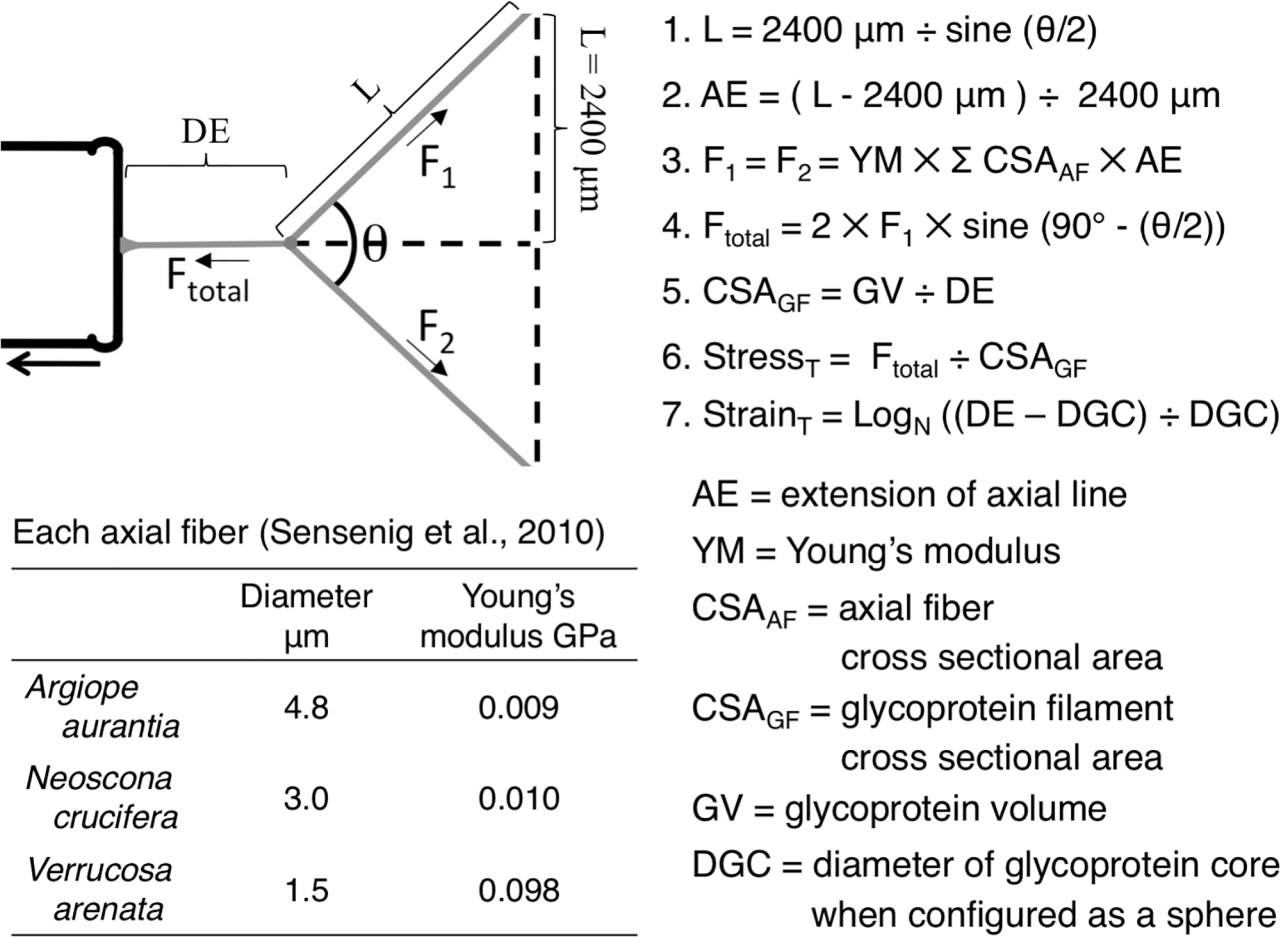
Values and formulas used to characterize glycoprotein performance and material properties.

The seven steps involved in determining the stress and strain of each viscous thread droplet at each of the four points during its extension are described in Fig 3. These include determining the: 1. Length of the elongated axial line on each side of the extended droplet as the hypotenuse of a right triangle, with the side opposite θ/2 being the initial, upstretched 2400 μm length, 2. Amount of axial line extension relative to the initial 2400 μm length each half of the axial line, 3. Force on the axial line as the result of this extension, determined from values for each of the paired axial line fibers provided in the table included in Fig 4, 4. Force on the extended droplet filament determined by resolving the force vectors of the two axial line halves, 5. Cross sectional area of the extended glycoprotein filament, determined by dividing the inferred volume of a droplet’s glycoprotein core by the extended droplet length, 6. True stress on the extended filament, determined by dividing the force on the extended glycoprotein filament by the filament’s cross sectional area and 7. True strain on the extended glycoprotein filament, expressed relative to the glycoprotein sphere’s initial diameter. We generated a stress strain curve for each species glycoprotein at each humidity using mean true stress and true strain values and from this determined Young’s modulus as the slope of the curve in its linear elastic phase. Toughness was computed as the area under these stress strain curves, as the sum of a series of rectangles centered at each mean stress-strain value. The Young’s modulus values of flagelliform fibers reported in the literature were measured at approximately 50% RH. We did not assess how changes in humidity affect this property. Although water is known to alter the properties of spider threads [51–53], at each of our experimental humidity a viscous thread’s flagelliform fibers as well as its glycoprotein core remained coated by the thread’s aqueous material.

### Confirming experimental procedures

Before deriving values from stress strain curves, we assessed the efficacy of our experimental procedures and evaluated droplet extensions to ensure that they met expectations and the assumptions of our modeling. S1–S3 Tables report experimental humidities, confirming that these closely matched target humidities. Even at full droplet extension flagelliform axial fibers on each side of a droplet did not extend more than 21% of their initial in-web lengths (S4 Table), indicating that flagelliform fibers were within their linear elastic region and could reliably be used to gauge the force on an extending droplet.

In a subsequent experiment, we examined the possibility that capture threads slipped through the carbon tape matrix as force was applied to a droplet thereby, compromising our method of determining the force on an extending glycoprotein filament. We compared the responses of threads attached only by carbon tape with thread to which we then applied a small drop of Elmer’s Glue^™^, a technique that has been shown to firmly secure threads [44, 54]. We did this at 30, 50, 70, and 90% RH for threads from the webs of six individuals of each species, measuring the axial line angle just before droplets began to extend, at 50% droplet extension, and at full extension. We also viewed movies with iMovie^™^ to determine the total time that the axial lines that supported an extending droplet was under tension as gauged by an angular deflection less than 180°. Comparisons of both the angles of axial line deflection and the durations of taut droplet extensions of the two treatments showed that only for *A*. *aurantia* threads at 90% RH was there evidence for thread slippage though tape, as denoted by a significant difference between the two treatments (S5 Table). Therefore, only *A*. *aurantia* threads at 90% RH results need be excluded from consideration.

### Addressing droplet formation on extending glycoprotein filaments

**Fig 5:**
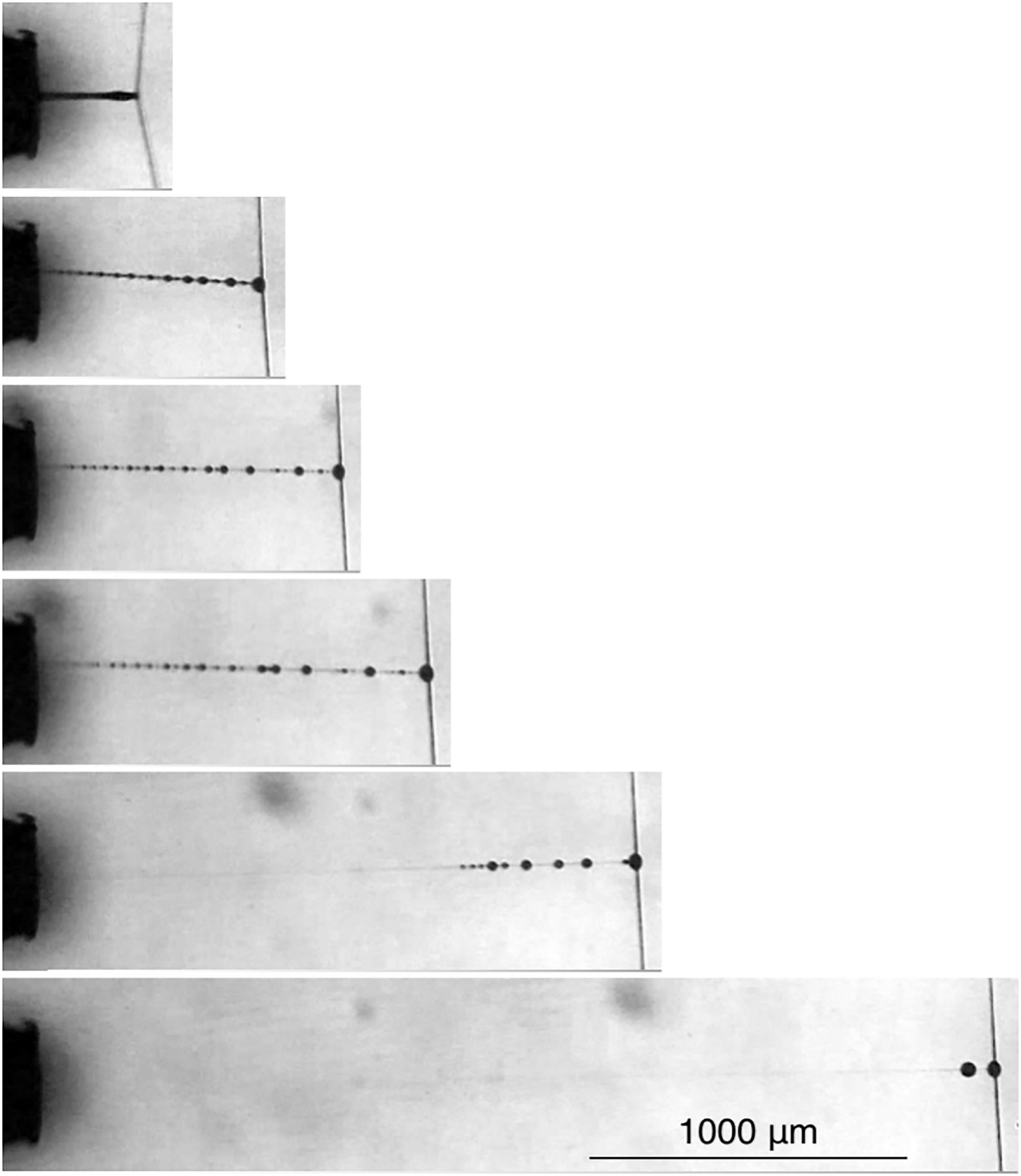
Progressive extension of an individual *A*. *aurantia* droplet, showing the formation of aqueous layer droplets on the glycoprotein filament.

**Fig 6:**
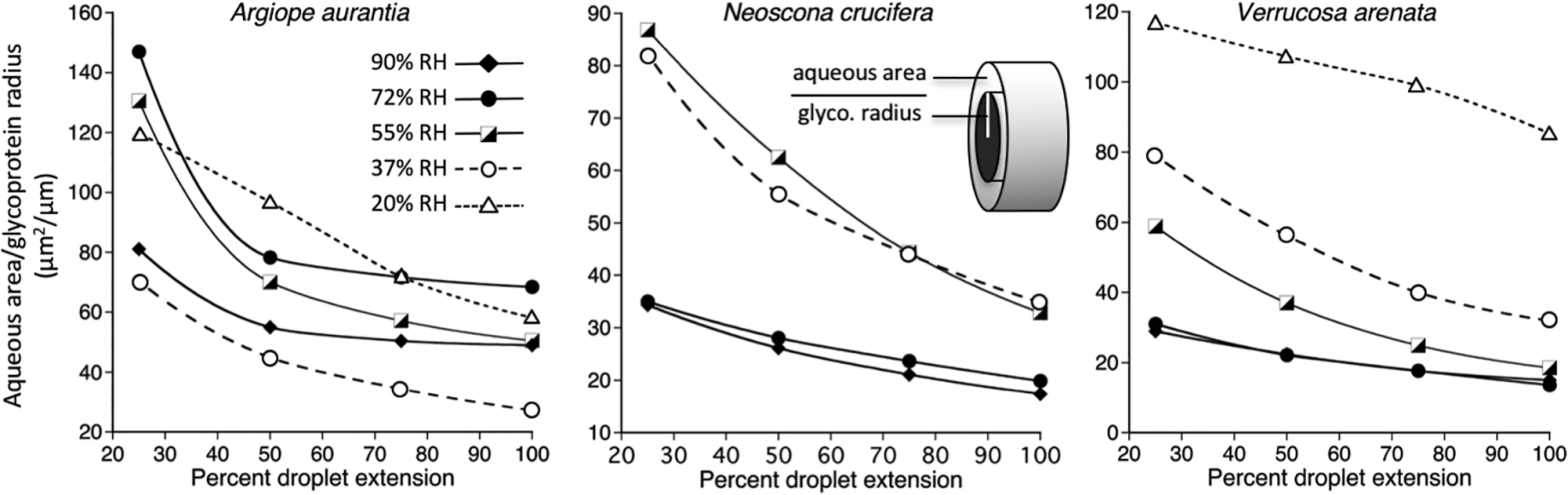
The effects of humidity and extension on the ratio of a glycoprotein filament’s circumference to the area of its aqueous layer. As surface tension is related to circumference, an increase in this index favors the formation of aqueous droplets like those shown in Fig 5.

When droplets of a thread span extend, their glycoprotein cores remain covered by aqueous material because their individual extensions are limited by stiffness of the thread’s flagelliform fibers (Fig 2). This was also the case of during the initial extension phase of the isolated, single droplets that we studied. However, as droplet extension progressed, the aqueous layer was progresses disrupted, exposing the glycoprotein (Fig 5). This was more evident at higher humidities, where reduced glycoprotein viscosity allowed longer droplet extensions. By drawing water away from the exposed glycoprotein regions and exposing them to evaporative loss, this disruption of the glycoprotein’s aqueous sheath has the potential to dry and stiffen glycoprotein. Moreover, glycoprotein is deprived of its plasticizing LMMCs [35]. Droplets appear to form on a filament to minimize the surface energy of the thinning aqueous sheath because these droplets have a lower surface to volume ratio than that of a continuous cylinder of aqueous material, a phenomenon described by Plateau-Rayleigh instability [19]. We evaluated this by computing a ratio of the circumference of what would have been a continuously aqueous layer coated glycoprotein filament divided by the cross-sectional area of the aqueous layer. We did this by dividing the volume of each component by the length of the extended droplet and positioning the glycoprotein filament in the center of the aqueous layer. As circumference is proportional to the aqueous layer’s surface tension, dividing this by aqueous volume yields an index whose increase is directly related to the instability of the aqueous layer and the likelihood that droplets will from on the glycoprotein filament. Plots show that this ratio increases both as humidity increases and as the length of an extending droplet increases (Fig 6).

**Fig 7:**
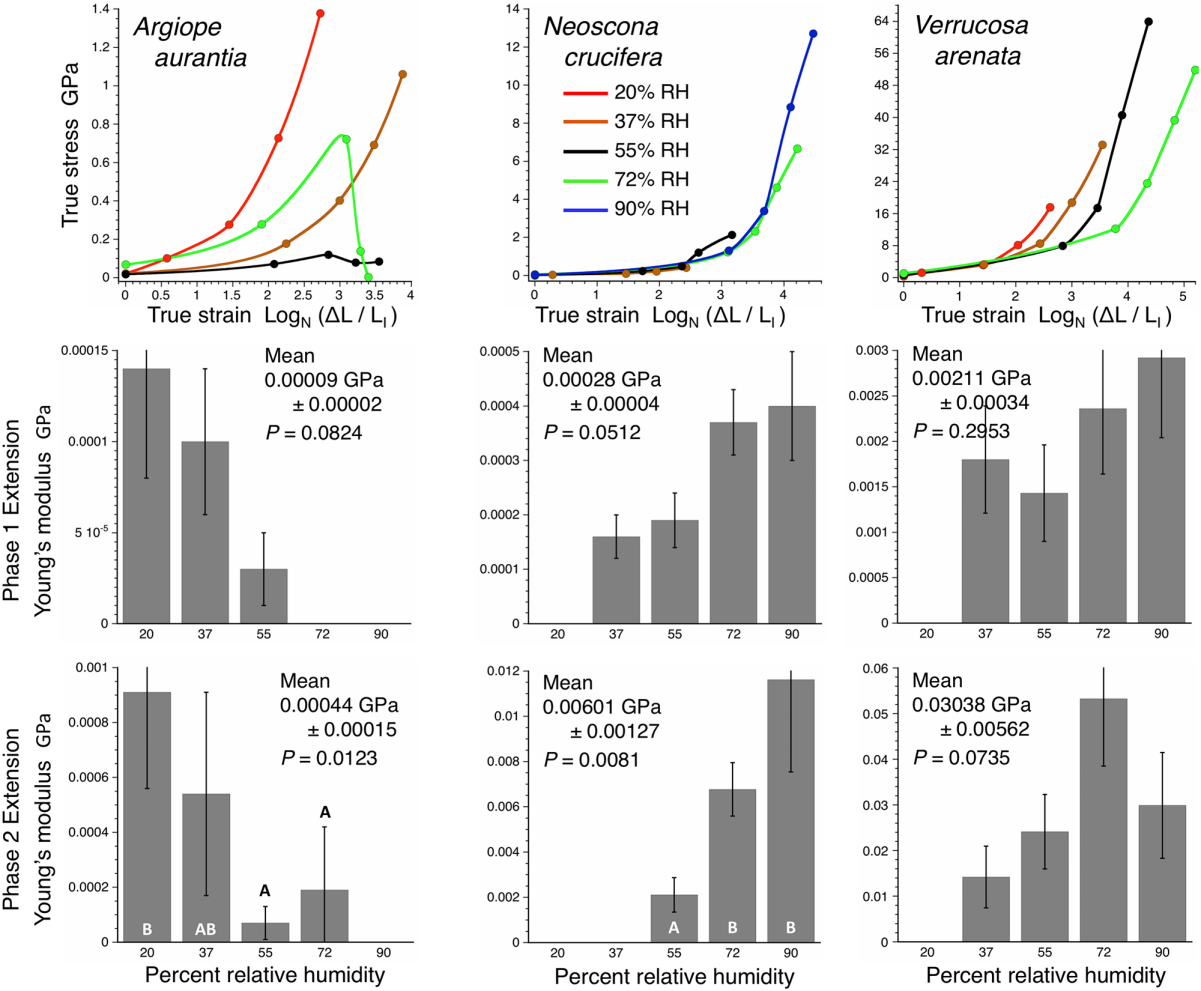
Stress-strain curves and Young’s modulus values derived from Phase 1 and Phase 2 region of stress-strain curves. Histogram error bars are ± 1 standard error. Grand means and standard errors are given for each species’ values and the *P* values of Wilcoxon Tests of the effect of RH on *E*. Letters denote the ranking of values that were significantly affected by RH, as determined by Wilcoxon Each Pairs Tests.

These observations and models indicate that the onset of droplet formation can be identified as a pronounced increase in the slope of a droplet’s stress-strain curve, a prediction that was supported (Fig 7). This signature allows us to identify two phases of droplet extension and to compute separate *E* and toughness values for each phase. Phase 1 occurs early in droplet extension when glycoprotein is fully enclosed in the aqueous layer and corresponds to the normal glycoprotein performance where droplet length is constringed by the stiffness of the thread’s flagelliform fibers (Fig 2). Phase 2 begins when portions of the glycoprotein filaments are exposed as droplets form. Table 1 identifies the limits of Phase 1 and Phase 2 portions of each species’ stress strain curves. Toughness values were separately computed for the areas under Phase 1 and Phase 2 regions of each stress-strain curve. Only *A*. *trifasciata* exhibited reliable values for 20% RH, as *Neoscona crucifera* droplets did not extend at 20% RH and only four *V*. *arenata* droplets extended at this humidity.

**Table 1:** Percent droplet extension ranges used for computing Young’s modulus (*E*) and percent droplet extension ranges summed to determine toughness (T) values for Phase 1 and Phase 2 of droplet extension.

	20% RH	37% RH	55% RH	72% RH	90% RH
*Argiope aurantia*					
*E* Phase 1	0–50%	0–25%	0–25%	---	---
*E* Phase 2	50–100%	50–100%	25–50%	25–50%	---
T Phase 1	25–50%	0–25%	0–25%	---	---
T Phase 2	50–100%	25–100	25–100%	0–100%	---
*Neoscona crucifera*					
*E* Phase 1	---	0–100%	0–50%	0–25%	0–25%
*E* Phase 2	---		50–100%	50–100%	50–100%
T Phase 1	---	0–100%	0–50%	0–25%	0–25%
T Phase 2	---	---	50–100%	25–100%	25–100
*Verrucosa arenata*					
*E* Phase 1	---	0–50%	0–25%	0–25%	0–25%
*E* Phase 2	---	50–100%	50–100%	50–100%	50–100%
T Phase 1	---	0–25%	0–25%	0–25%	0–25%
T Phase 2	---	25–100	25–100%	25–100%	25–100%

## Results

**Fig 8:**
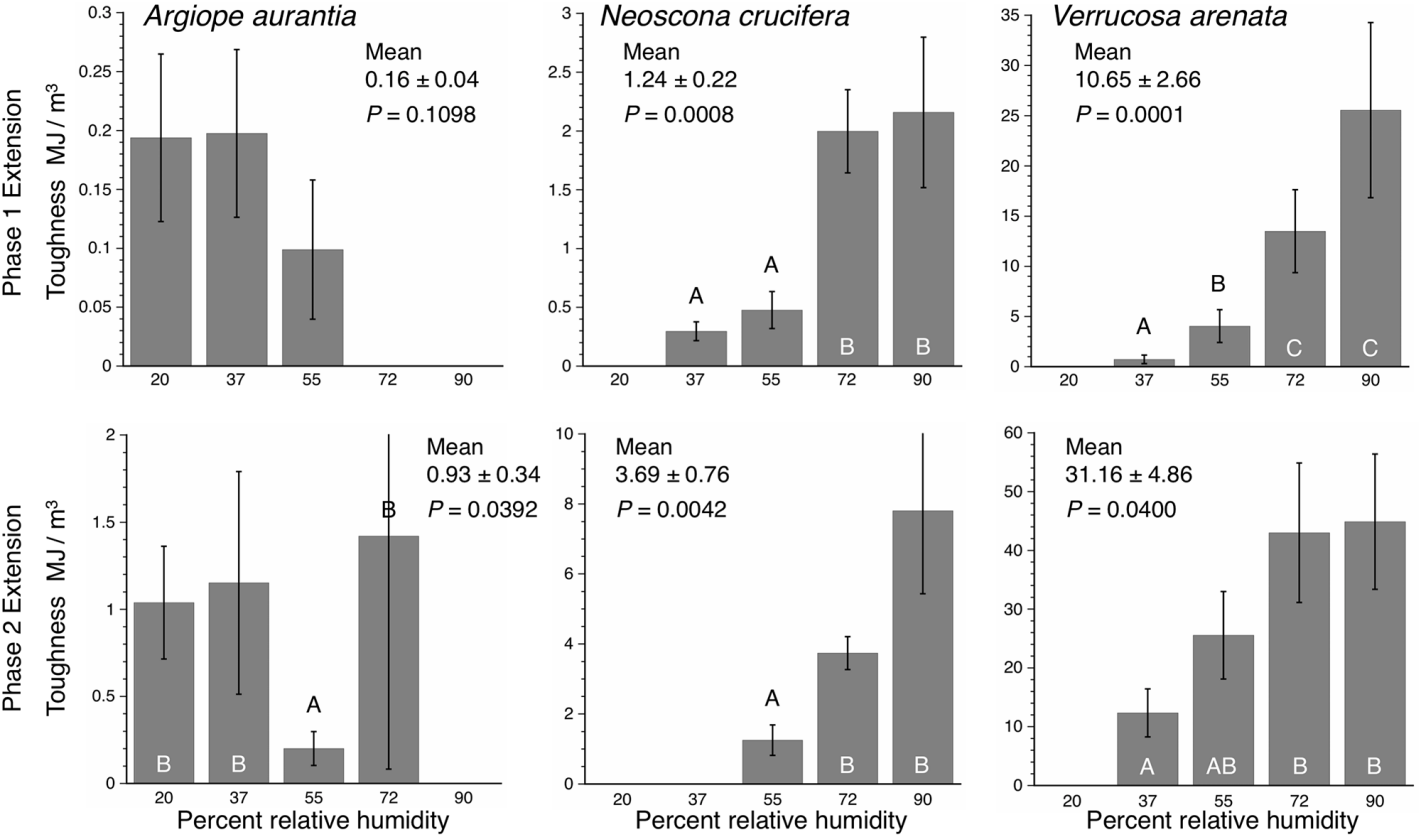
Toughness values derived from Phase 1 and Phase 2 region of stress-strain curves. Histogram error bars are ± 1 standard error. Grand means and standard errors are given for each species’ values and the *P* values of Wilcoxon Tests of the effect of RH. Letters denote the ranking of values that were significantly affected by RH, as determined by Wilcoxon Each Pairs Tests.

S6–S8 Tables summarize the three species’ axial line deflections, computed forces on extended droplets, and droplet lengths from 25%—full extensions. S9 Table provides Phase 1 and Phase 2 stress, strain, Young’s modulus, and toughness values for all individuals of each species at each RH. In all intra-specific and inter-specific comparisons of *E* and toughness most values were not normally distributed (Shapiro-Wilk W Tests *P* ≤ 0.05). Therefore, we used Wilcoxon Tests to identify differences in values and, when differences were identified, Wilcoxon Each Pair Tests to rank values, considering *P* ≤ 0.05 to be significant. Figs 7 and 8 compare the effect of RH on the *E* and toughness values of the three species glycoproteins. In *A*. *aurantia* both Phase 1 and Phase 2 *E* decreased with humidity, although this was significant only for Phase 2 values. In *N*. *crucifera*, both Phase 1 and Phase 2 *E* increased with humidity, although Phase 1 differences were only marginally significant. *Verrucosa arenata E* did not differ significantly, with Phase 1 values showing little change and Phase 2 values increasing to 72% RH and then decreased. In *A*. *aurantia*, only Phase 2 toughness differed with humidity, a result that can be attributed to a very low 55% RH value. Both Phase 1 and Phase 2 glycoprotein toughness increased with humidity in *N*. *crucifera* and *V*. *arenata*.

For humidities at which *E* and toughness could be reliably assessed these values showed an increase from *A*. *aurantia* to *N*. *crucifera* to *V*. *arenata* (Tables 2 and 3). Phase 2 *E* and toughness values were greater that Phase 1 values and in most cases Matched Pairs Tests showed this difference to be significant (*P* ≤ 0.05) (Tables 4 and 5).

**Table 2:** Inter-specific comparisons of Young’s modulus values.

	37% RH GPa		55% RH GPa		72% RH GPa		90% RH GPa	
	Phase 1	Phase 2	Phase 1	Phase 2	Phase 1	Phase 2	Phase 1	Phase 2
*Argiope aurantia*								
	A	A	A	A		A		
*Neoscona crucifera*								
	AB		B	B	A	B	A	
*Verrucosa arenata*								
	C	B	C	C	B	C	B	
Wilcoxon *P*	0.0001	0.0001	0.0001	0.0001	0.0046	0.0001	0.0015	0.8919

Letters A–C identify inter-specific ranking (low to high values) of Young’s modulus as determined by Wilcoxon Each Pairs Tests.

**Table 3:** Inter-specific comparisons of toughness values.

	37% RH GPa		55% RH GPa		72% RH GPa		90% RH GPa	
	Phase 1	Phase 2	Phase 1	Phase 2	Phase 1	Phase 2	Phase 1	Phase 2
*Argiope aurantia*								
		A	A	A		A		
*Neoscona crucifera*								
			B	B	A	B	A	A
*Verrucosa arenata*								
		B	C	C	B	C	B	B
Wilcoxon *P*	0.5794	0.0002	0.0001	0.0001	0.0147	0.0001	0.0004	0.0209

Letters A–C identify inter-specific ranking (low to high values) of Young’s modulus as determined by Wilcoxon Each Pairs Tests.

**Table 4:** Intra-specific comparisons of Phase 1 and Phase 2 Young’s modulus values.

	20% RH	37% RH	55% RH	72% RH	90% RH
*Argiope aurantia*					
	**0.0251**	0.2355	0.3566		
*Neoscona crucifera*					
			**0.0103**	**0.0002**	**0.0150**
*Verrucosa arenata*					
		**0.0365**	**0.0067**	**0.0020**	**0.0204**

*P* values of Matched Pairs Tests.

**Table 5:** Intra-specific comparisons of Phase 1 and Phase 2 toughness values.

	20% RH	37% RH	55% RH	72% RH	90% RH
*Argiope aurantia*					
	**0.0077**	0.0823	0.1654		
*Neoscona crucifera*					
			**0.0418**	**0.0027**	**0.0068**
*Verrucosa arenata*					
		**0.0117**	**0.0055**	**0.0044**	**0.0247**

*P* values of Matched Pairs Tests.

**Fig 9:**
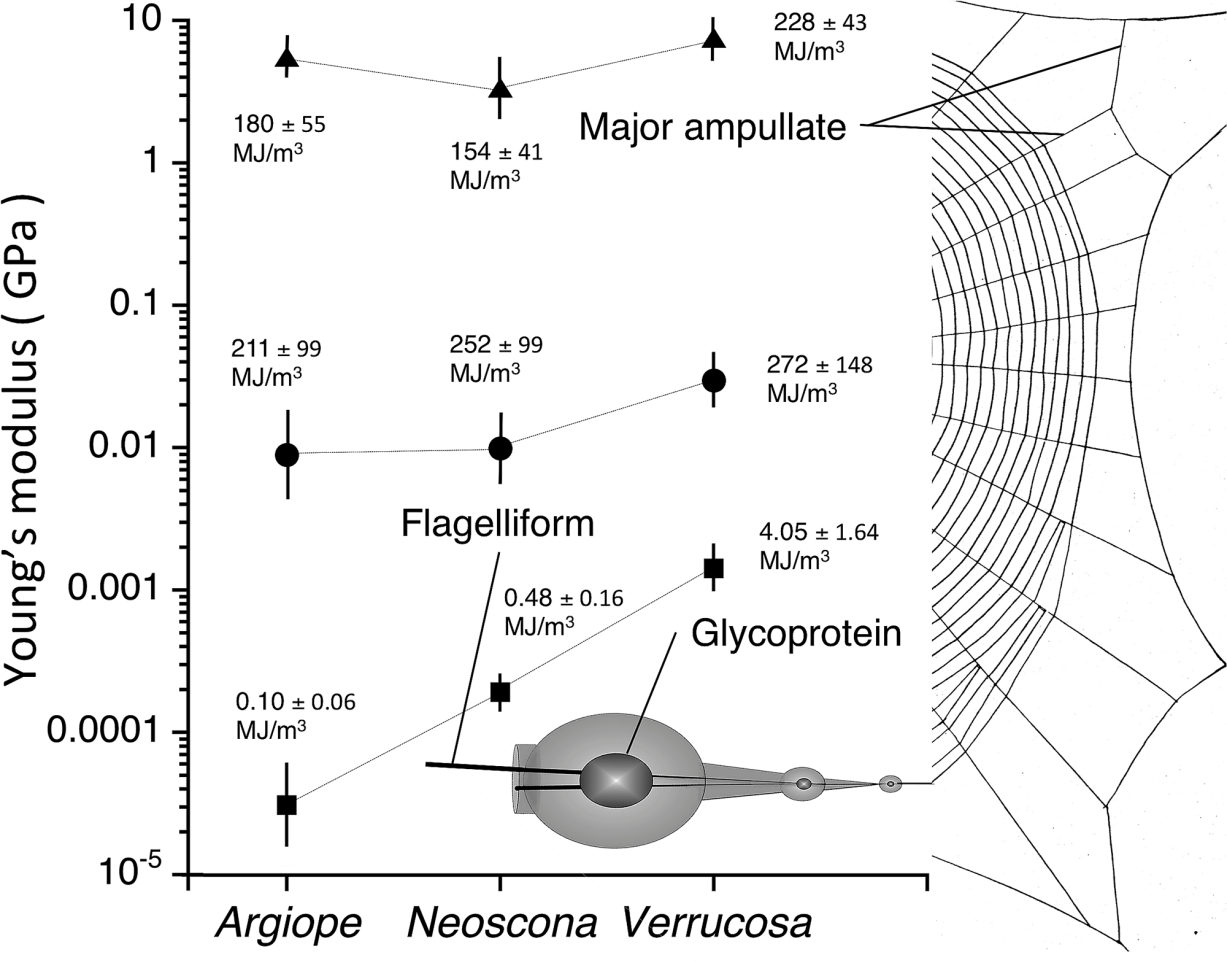
Comparison of 55% RH Phase 1 Young’s modulus and toughness of glycoprotein from this study with flagelliform, and major ampullate fiber values measured in the range of 50% RH (Sensenig et al., 2010). Young’s modulus values are plotted and described on the Y-axis. Toughness values appear beside these points. Error bars are ± 1 standard error. Lines connecting the glycoprotein, flagelliform, and major ampullate values are provided to make it easier to interpret the figure and are not regression lines.

Fig 9 compares the material properties of the three species’ glycoproteins, flagelliform fibers, and major ampullate threads. This small number of species does now support phylogenetic comparative methods and statistical tests of relationships among these values can only be viewed as suggestive. The only significant correlation among the web element’s *E* and toughness values was between glycoprotein and flagelliform fiber Young’s modulus (r = 0.998, P = 0.0319), which lends tentative support to the coordinated evolution of the glycoprotein and axial fiber stiffness.

## Discussion

As hypothesized, when compared at the same humidity, the Young’s modulus of the three species’ glycoproteins was less than that of their capture thread’s flagelliform fibers (Fig 9). Flagelliform *E* ranged from 0.01 to 0.03 GPa [12], whereas Phase 1 glycoprotein *E* ranged from 0.00003 to 0.00140 GPa. Flagelliform values were 21, 52, and 300 times greater than Phase 1 glycoprotein values in *V*. *arenata*, *N*. *crucifer*, and *A*. *arenata*, respectively. Flagelliform fiber toughness ranged from 211–272 MJ/m^3^ [12], whereas Phase 1 glycoprotein toughness ranged from 0.10–4.05 MJ/m^3^. Flagelliform fiber toughness was 67, 525, and 2110 times greater than Phase 1 glycoprotein toughness in *V*. *arenata*, *N*. *crucifera*, and *A*. *arenata*, respectively. A fuller understanding of the scaling glycoprotein and flagelliform fiber *E* and its importance for the synergistic integration of capture thread components will require both a larger taxon sample and models that also incorporate data on droplet distribution, glycoprotein volume, droplet extension, and flagelliform diameter.

The apparent lack of association between *E* of major ampullate threads and the capture thread’s components is not surprising, given the different roles that these web components play in prey capture. Major ampullate threads form the web’s radii and function to absorb and dissipate the force when an insect strikes an orb web [13, 14]. Therefore, major ampullate threads have evolved as stiff elements. Largely freed from that task, capture threads have evolved as more compliant web elements. This allows them to conform to an insect’s surface and stretch as the insect struggles to escape. The extension of both the flagelliform fibers and glycoprotein serves two purposes: it facilitates a suspension bridge mechanism that is responsible for summing the adhesive forces of multiple thread droplets [26] and it dissipates the energy of the insect’s struggles [43].

When a droplet’s glycoprotein is contained within its aqueous layer (Phase 1 extension), as typical during the performance of capture thread strands, *E* differs greatly among species (Fig 7), with *N*. *crucifera* E being 3.1 times that of *A*. *aurantia* and *V*. *arenata E* being 7.5 times that of *N*. *crucifera*. In this native state values range from 0.00003 GPa, which is similar to the *E* of fibronectin, a glycoprotein that anchors cells in the extracellular matrix [55] to 0.00292 GPa, a value similar to that of resilin in insect ligaments [56]. The ranges of Phase 1 glycoprotein *E* exhibited by *V*. *arenata* and *N*. *crucifera* were small (0.0014–0.0029 and 0.00016–0.00040 GPa, respectively), but that exhibited by *A*. *aurantia* was greater (0.00003–0.00014 GPa) (Fig 7). Although not statistically significant, these intra-specific differences suggest that glycoprotein stiffness responded to humidity.

Orb spider glycoprotein remains extensible after droplets form on the extending glycoprotein filament (Phase 2 extension). However, the glycoprotein stiffens during this phase because it was increasingly exposed to the drying effects of air. It was also deprived of LMMCs in the aqueous layer, which plasticize the glycoprotein [35]. When the aqueous layer was experimentally removed, glycoprotein adhesion at 100% RH was only half that of native droplets [35]. Moreover, Cross Polarization Magic Angle Spinning used in that study revealed that, at the molecular level, glycoprotein became more rigid and an unresponsive to changes in humidity when deprived of LMMCs. This may account for Phase 2 *E* being 4.7 times that of Phase 1 *E* in *A*. *aurantia*, 19.4 times greater in *N*. *crucifera*, and 19.2 times greater in *V*. *arenata*. Phase 2 E ranging from 0.00007 to 0.053 GPa, extending glycoprotein stiffness into the range of the proximal region of mussel threads fibers [57] and the mid-range of silicone rubber [58]. Native glycoprotein toughness ranges from 0.1 MJ/m^3^ at 55% RH in *A*. *aurantia* to 26 MJ/m^3^ at 90% RH in *V*. *arenata*. In these species and at these humidities Phase 2 toughness ranges from 0.2 to 45 MJ/m^3^.

## Conclusions

Compounds in the aqueous material that surrounds the adhesive glycoprotein cores of capture thread droplets ensures that the glycoprotein is hydrated, maintains glycoprotein integrity, and ensures glycoprotein extensibility. This extensibility combines with that of a thread’s axial lines to dissipate the force of a struggling prey and sum the adhesion of multiple droplets. Our study documents the broad range of orb spider glycoprotein elastic modulus and toughness and shows that these proteins continue to exhibit desirable properties outside of their native aqueous covering. Humidity affects the material properties that a species’ glycoprotein expresses, but the greatest difference is seen among species. The elastic modulus of a species’ glycoprotein is much less than that of a capture thread’s supporting flagelliform fibers. In our limited sample of three species, stiffer axial lines are associated with stiffer glycoproteins, suggesting that the properties of viscous capture thread components may have evolved in a synergistic fashion to optimize thread adhesion, a hypothesis that remains to be tested.

## Supporting information

S1 Fig Changes in glycoprotein volume with increasing relative humidity. When *Verrucosa arenata* volume is computed in the same manner as *Neoscona crucifera* and *Argiope aurantia* its high viscosity produces erroneous results, making it appear that volume decreases precipitously as humidity increases. When *V*. *arenata* glycoprotein volume is adjusted, these corrected values show the same trend as the other two species.
(TIF)

S2 Fig Flattened droplet thickness, indicating a decrease in viscosity with increasing humidity. Mean ± 1 standard error. At 72% and 90% RH the standard error bars of *V*. *arenata* are hidden by its symbols.
(TIF)

S1 Table Features of *Argiope aurantia* droplets and the humidities at which they were measured (mean ± 1 standard error). Sample size: 20% RH, 37% RH, and 55% RH = 14; 72% RH = 11; 90% = 13.
(DOCX)

S2 Table Features of *Neoscona crucifera* droplets and the humidities at which they were measured (mean ± 1 standard error). Sample size: 37% RH = 5; 55% RH = 13; 72% RH = 12; 90% RH = 14.
(DOCX)

S3 Table Features of *Verrucosa arenata* droplets and the humidities at which they were measured (mean ± 1 standard error). Sample size: 20% RH, = 11; 37% RH, 55% RH, 72% RH, and 90% RH = 12.
(DOCX)

S4 Table Ranges of individual spider mean percent axial line extension at sampled droplet extension phases, with standard error of the largest value indicated. (DOCX)

S5 Table Comparison of axial line angles at droplet release and total loaded extension times between threads that were secured to sampler supports by carbon tape alone and those to which Elmer’s^®^ glue was subsequently added. Angles area compared at pre-extension, just prior to droplet extension, and at maximum extension. Mean ± 1 standard error, T = *t* test for normally distributed values; W = Wilcoxon test for values that were not normally distributed.
(DOCX)

S6 Table *Argiope aurantia* axial line deflection, computed force on extended droplet, and droplet length from 25%—Full extension. Mean ± 1 standard error.
(DOCX)

S7 Table *Neoscona crucifera* axial line deflection, computed force on extended droplet, and droplet length from 25%—Full extension. Mean ± 1 standard error.
(DOCX)

S8 Table *Verrucosa arenata* axial line deflection, computed force on extended droplet, and droplet length from 25%—Full extension. Mean ± 1 standard error.
(DOCX)

S9 Table Excel^®^ table of Phase 1 and Phase 2 stress, strain, Young’s modulus, and toughness values for each test humidity. (XLSX)

S1 Photo An *Argiope aurantia* female attaches a capture thread to a radial line as she completes her orb web’s capture spiral. In the early morning’s high humidity the glue droplets of previously deposited capture threads have attracted atmospheric moisture and enlarged.
(JPG)
