# Supplementary figures and images for: Elastic modulus and toughness of orb spider glycoprotein glue
Source: PLoS One. 2018 May 30;13(5):e0196972. doi: 10.1371/journal.pone.0196972 (PMC5976159; doi:10.1371/journal.pone.0196972)

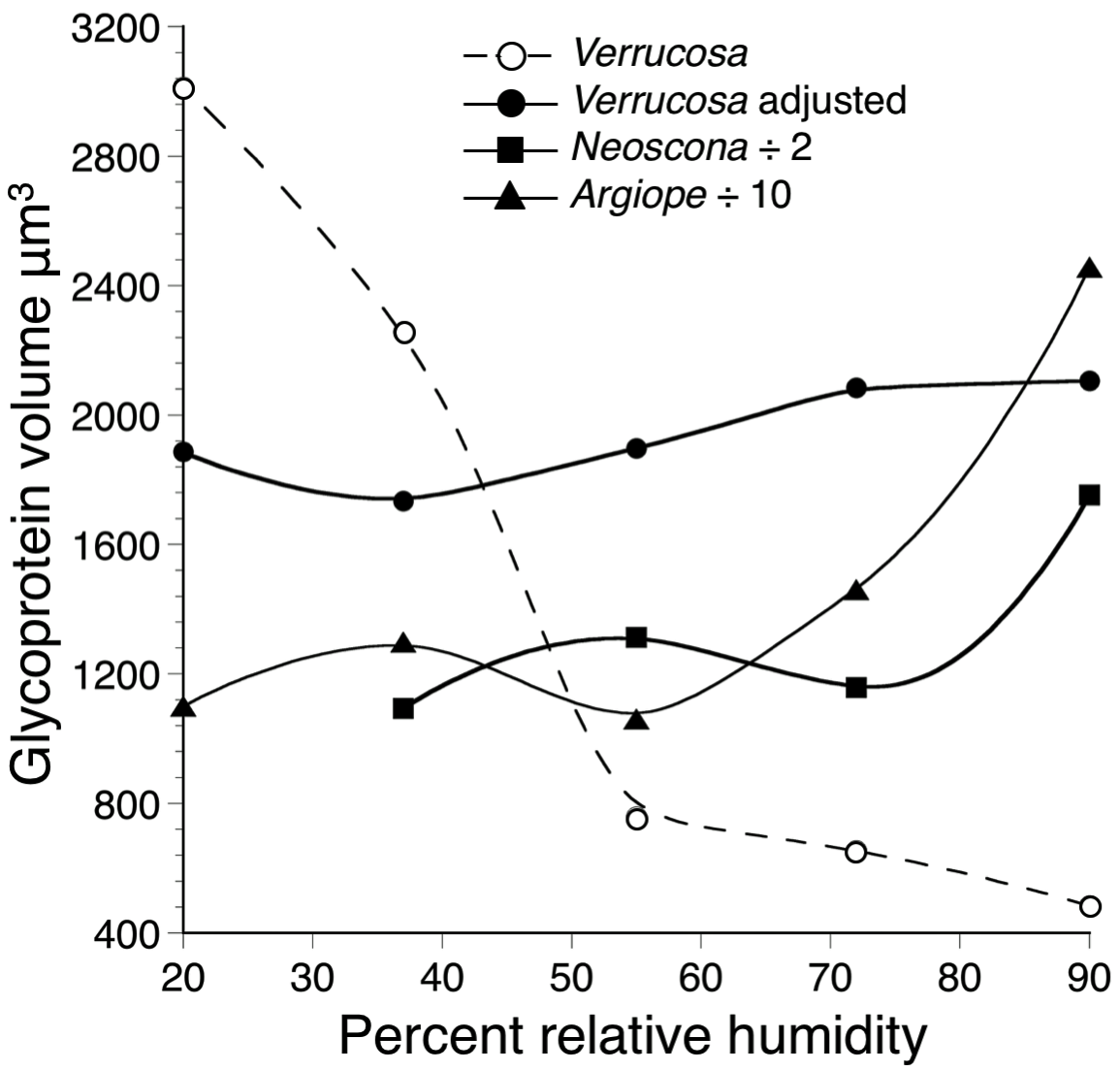

Supplement: S1 Fig — When Verrucosa arenata volume is computed in the same manner as Neoscona crucifera and Argiope aurantia its high viscosity produces erroneous results, making it appear that volume decreases precipitously as humidity increases. When V. arenata glycoprotein volume is adjusted, these corrected values show the same trend as the other two species. (TIF) [file pone.0196972.s001.tif]

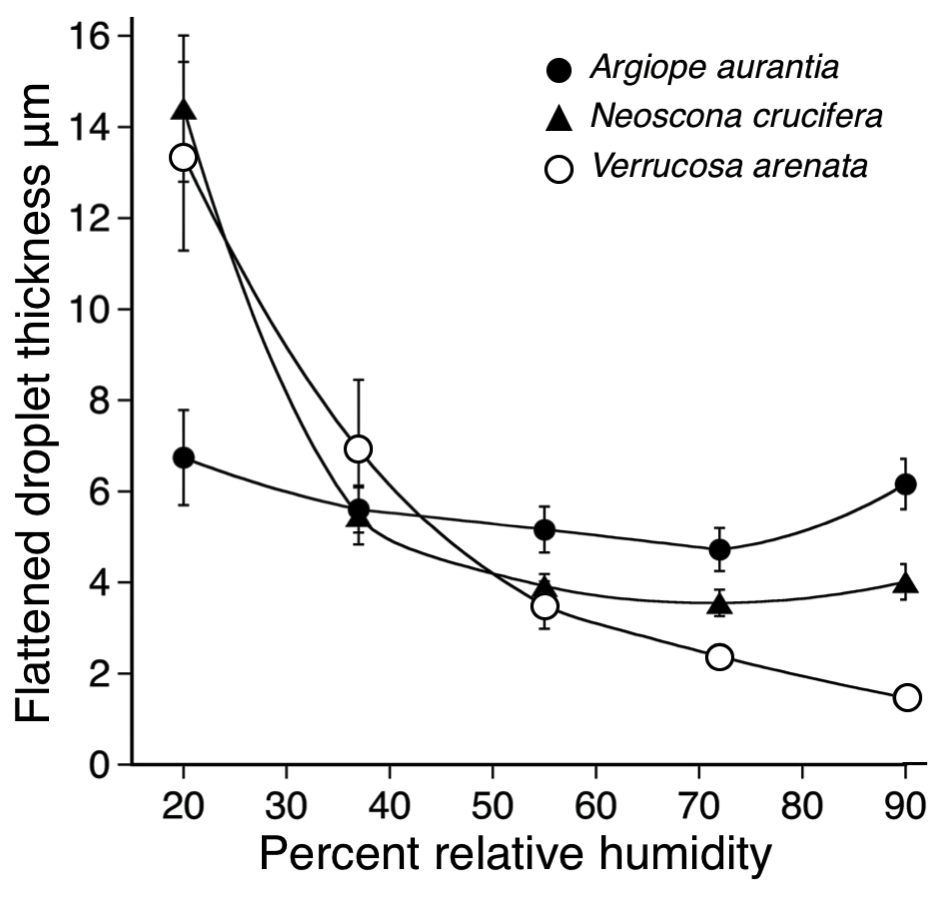

Supplement: S2 Fig — Mean ± 1 standard error. At 72% and 90% RH the standard error bars of V. arenata are hidden by its symbols. (TIF) [file pone.0196972.s002.tif]

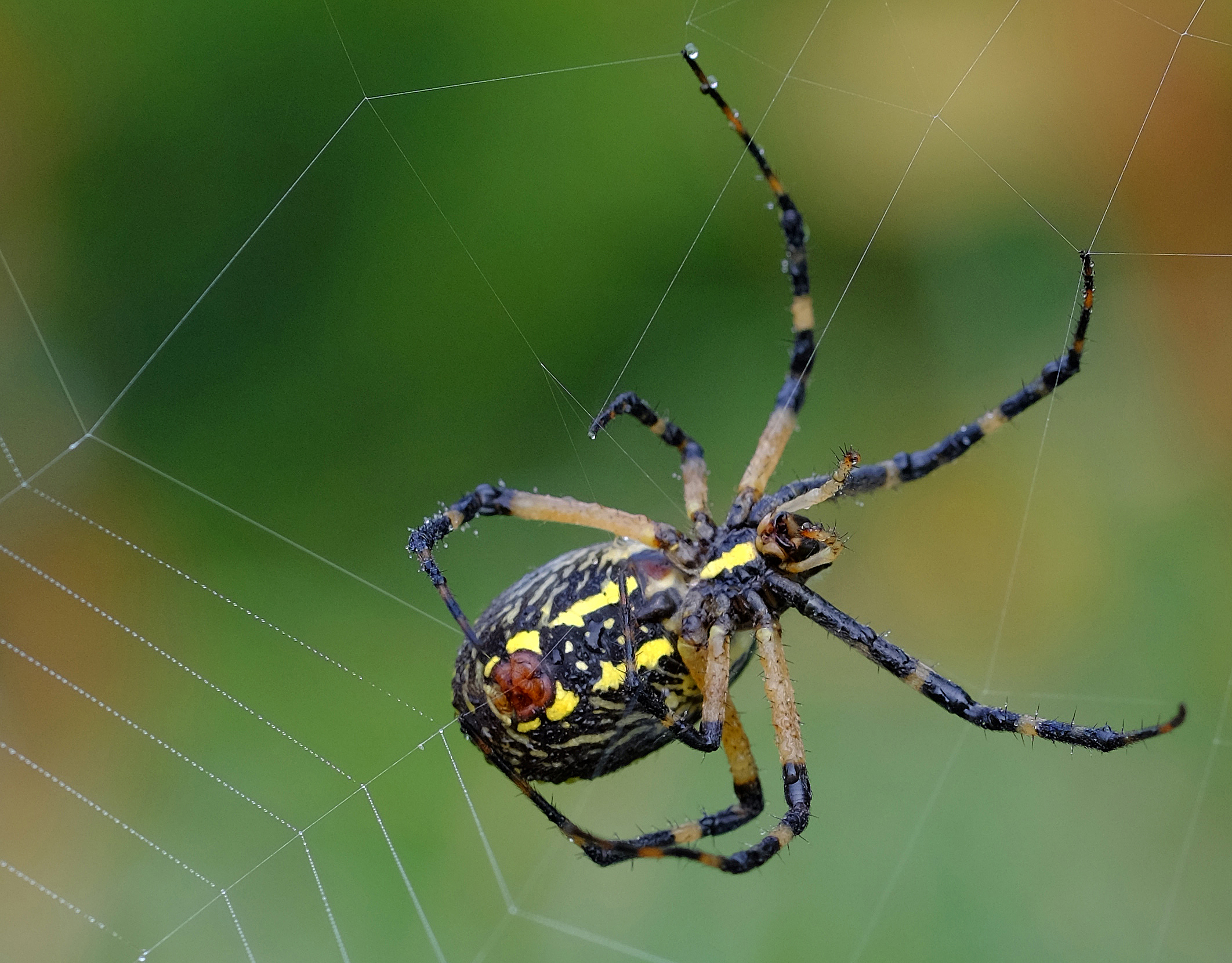

Supplement: S1 Photo — In the early morning’s high humidity the glue droplets of previously deposited capture threads have attracted atmospheric moisture and enlarged. (JPG) [file pone.0196972.s012.jpg]
