# Supplementary material for: Elastic modulus and toughness of orb spider glycoprotein glue
Source: PLoS One. 2018 May 30;13(5):e0196972. doi: 10.1371/journal.pone.0196972 (PMC5976159; doi:10.1371/journal.pone.0196972)
Supplement: S1 Table — Sample size: 20% RH, 37% RH, and 55% RH = 14; 72% RH = 11; 90% = 13. (DOCX) [file pone.0196972.s003.docx]

**S1 Table. Features of *Argiope aurantia* droplets and the humidities at which they were measured.** Mean ± 1 standard error. Sample size: 20% RH, 37% RH, and 55% RH = 14; 72% RH = 11; 90% = 13.

| Relative Humidity | 20% | 37% | 55% | 72% | 90% |
| --- | --- | --- | --- | --- | --- |
| **Humidity** |  |  |  |  |  |
| Suspended | 20.5 ± 0.3 | 37.4 ± 0.1 | 55.1 ± 0.1 | 72.1 ± 0.2 | 90.2 ± 0.3 |
| Flattened | 20.7 ± 0.2 | 37.2 ± 0.1 | 55.5 ± 0.1 | 71.9 ± 0.2 | 90.5 ± 0.3 |
| Extended | 20.5 ± 0.4 | 36.6 ± 0.2 | 55.1 ± 0.1 | 71.9 ± 0.1 | 90.0 ± 0.0 |
| **Droplet** |  |  |  |  |  |
| Length µm | 61 ± 4 | 64 ± 3 | 64 ± 4 | 67 ± 5 | 80 ± 5 |
| Width µm | 44 ± 3 | 47 ± 2 | 49 ± 4 | 49 ± 4 | 60 ± 4 |
| Volume µm^3^ | 56009 ± 9623 | 65590 ± 10232 | 71139 ± 17186 | 78806 ± 17186 | 137851 ± 23796 |
| Flat area µm^2^ | 9193 ± 1582 | 11217 ± 1111 | 12964 ± 1705 | 16968 ± 3290 | 21450 ± 2910 |
| **Glycoprotein** |  |  |  |  |  |
| Flat area µm^2^ | 1352 ± 210 | 1639 ± 148 | 1502 ± 195 | 2213 ± 469 | 3231 ± 410 |
| Volume µm^3^ | 9030 ± 1978 | 9968 ± 1774 | 8625 ± 1586 | 10693 ± 2746 | 21515 ± 4088 |
| Glycoprotein Ratio | 0.153 ± 0.009 | 0.152 ± 0.010 | 0.119 ± 0.008 | 0.128 ± 0.009 | 0.153 ± 0.006 |
| **Extension** |  |  |  |  |  |
| Length µm | 64 ± 3 | 67 ± 4 | 68 ± 4 | 73 ± 5 | 84 ± 5 |
| Width µm | 48 ± 3 | 50 ± 3 | 51 ± 3 | 55 ± 4 | 65 ± 3 |
| Drop vol. µm^3^ | 68875 ± 11476 | 82082 ± 16258 | 86126 ± 16474 | 106789 ± 23062 | 165318 ± 28308 |
| Inf. glyco. vol. µm^3^ | 11002 ± 2180 | 12973 ± 2424 | 10597 ± 2350 | 14680 ± 4189 | 24564 ± 4311 |
