# Supplementary material for: Elastic modulus and toughness of orb spider glycoprotein glue
Source: PLoS One. 2018 May 30;13(5):e0196972. doi: 10.1371/journal.pone.0196972 (PMC5976159; doi:10.1371/journal.pone.0196972)
Supplement: S2 Table — Sample size: 37% RH = 5; 55% RH = 13; 72% RH = 12; 90% RH = 14. (DOCX) [file pone.0196972.s004.docx]

**S2 Table. Features of *Neoscona crucifera* droplets and the humidities at which they were measured.** Mean ± 1 standard error. Sample size: 37% RH = 5; 55% RH = 13; 72% RH = 12; 90% RH = 14.

| Relative Humidity | 20% | 37% | 55% | 72% | 90% |
| --- | --- | --- | --- | --- | --- |
| **Humidity** |  |  |  |  |  |
| Suspended | -- | 37.2 ± 0.2 | 55.2 ± 0.2 | 71.9 ± 0.3 | 90.1 ± 0.1 |
| Flattened | -- | 36.8 ± 0.4 | 55.2 ± 0.2 | 72.1 ± 0.2 | 90.0 ± 0.1 |
| Extended | -- | 36.8 ± 0.2 | 55.3 ± 0.3 | 71.8 ± 0.3 | 90.1 ± 0.1 |
| **Droplet** |  |  |  |  |  |
| Length µm | -- | 38 ± 1 | 38 ± 2 | 41 ± 2 | 43 ± 2 |
| Width µm | -- | 29 ± 1 | 28 ± 1 | 32 ± 1 | 33 ± 1 |
| Volume µm^3^ | --- | 13264 ± 958 | 13378 ± 1716 | 18739 ± 2599 | 20213 ± 2229 |
| Flat area µm^2^ | -- | 2512 ± 197 | 3312 ± 329 | 5059 ± 508 | 5273 ± 603 |
| **Glycoprotein** |  |  |  |  |  |
| Flat area µm^2^ | -- | 321 ± 64 | 395 ± 50 | 539 ± 50 | 572 ± 79 |
| Volume µm^3^ | -- | 1726 ± 372 | 1642 ± 268 | 2007 ± 267 | 2219 ± 312 |
| Glycoprotein Ratio | -- | 0.127 ± 0.022 | 0.116 ± 0.007 | 0.108 ± 0.004 | 0.108 ± 0.006 |
| **Extension** |  |  |  |  |  |
| Length µm | -- | 38 ± 2 | 43 ± 2 | 43 ± 3 | 49 ± 3 |
| Width µm | -- | 28 ± 2 | 32 ± 2 | 33 ± 2 | 38 ± 2 |
| Drop vol. µm^3^ | -- | 13446 ± 2337 | 20646 ± 4336 | 22716 ± 5405 | 32671 ± 5240 |
| Inf. glyco. vol. µm^3^ | -- | 2189 ± 589 | 2625 ± 696 | 2315 ± 454 | 3504 ± 597 |
