# Supplementary material for: Elastic modulus and toughness of orb spider glycoprotein glue
Source: PLoS One. 2018 May 30;13(5):e0196972. doi: 10.1371/journal.pone.0196972 (PMC5976159; doi:10.1371/journal.pone.0196972)
Supplement: S3 Table — Sample size: 20% RH, = 11; 37% RH, 55% RH, 72% RH, and 90% RH = 12. (DOCX) [file pone.0196972.s005.docx]

**S3 Table. Features of *Verrucosa arenata* droplets and the humidities at which they were measured.** Mean ± 1 standard error. Sample size: 20% RH, = 11; 37% RH, 55% RH, 72% RH, and 90% RH = 12.

| Relative Humidity | 20% | 37% | 55% | 72% | 90% |
| --- | --- | --- | --- | --- | --- |
| **Humidity** |  |  |  |  |  |
| Suspended | 20.0 ± 0.0 | 37.0 ± 0.0 | 55.0 ± 0.0 | 72.0 ± 0.0 | 90.0 ± 0.0 |
| Flattened | 20.0 ± 0.0 | 37.0 ± 0.1 | 55.0 ± 0.0 | 72.0 ± 0.0 | 90.0 ± 0.0 |
| Extended | 20.0 ± 0.0 | 37.0 ± 0.0 | 55.0 ± 0.0 | 72.0 ± 0.0 | 90.0 ± 0.0 |
| **Droplet** |  |  |  |  |  |
| Length µm | 32 ± 3 | 31 ± 2 | 34 ± 2 | 34 ± 2 | 35 ± 2 |
| Width µm | 23 ± 2 | 22 ± 2 | 26 ± 1 | 26 ± 2 | 27 ± 2 |
| Volume µm^3^ | 8366 ± 1690 | 7822 ± 1544 | 10561 ± 1672 | 10926 ± 1875 | 12649 ± 2360 |
| Flat area µm^2^ | 644 ± 70 | 1339 ± 250 | 3088 ± 376 | 4330 ± 461 | 8328 ± 1280 |
| **Glycoprotein** |  |  |  |  |  |
| Flat area µm^2^ | 168 ± 34 | 167 ± 35 | 206 ± 30 | 247 ± 40 | 284 ± 47 |
| Volume µm^3^ | 2412 ± 630 | 1479 ± 569 | 821 ± 230 | 680 ± 175 | 443 ± 89 |
| Glycoprotein Ratio | 0.255 ± 0.033 | 0.174 ± 0.057 | 0.069 ± 0.010 | 0.055 ± 0.006 | 0.034 ± 0.002 |
| **Extension** |  |  |  |  |  |
| Length µm | 34 ± 2 | 34 ± 3 | 33 ± 2 | 34 ± 2 | 37 ± 1 |
| Width µm | 25 ± 2 | 26 ± 2 | 25 ± 2 | 27 ± 2 | 29 ± 1 |
| Drop vol. µm^3^ | 10392 ± 1882 | 11527 ± 2339 | 10282 ± 1691 | 11338 ± 1807 | 14403 ± 1599 |
| Inf. glyco. vol. µm^3^ | 3013 ± 827 | 2257 ± 939 | 760 ± 168 | 655 ± 136 | 484 ± 51 |
