# Supplementary material for: Elastic modulus and toughness of orb spider glycoprotein glue
Source: PLoS One. 2018 May 30;13(5):e0196972. doi: 10.1371/journal.pone.0196972 (PMC5976159; doi:10.1371/journal.pone.0196972)
Supplement: S4 Table — (DOCX) [file pone.0196972.s006.docx]

**S4 Table. Ranges of mean axial line extension percentages at sampled droplet extension phases, with standard error of the largest value indicated.**

| Extension | *Argiope aurantia* | *Neoscona crucifera* | *Verrucosa arenata* |
| --- | --- | --- | --- |
| 25% | 1.69-4.32 ± 0.87 | 1.58-5.43 ± 0.60 | 4.58-16.63 ± 3.33 |
| 50% | 1.375-5.690 ± 1.14 | 2.051-7.153 ± 0.79 | 4.995-19.719 ± 4.12 |
| 75% | 0.049-6.941 ± 1.53 | 2.771-8.701 ± 0.88 | 5.372-21.382 ± 4.78 |
| 99% | 0.015-6.949 ± 1.73 | 3.32-9.318 ± 1.07 | 5.863-21.444 ± 2.73 |
