# Supplementary material for: Elastic modulus and toughness of orb spider glycoprotein glue
Source: PLoS One. 2018 May 30;13(5):e0196972. doi: 10.1371/journal.pone.0196972 (PMC5976159; doi:10.1371/journal.pone.0196972)
Supplement: S5 Table — Angles area compared at pre-extension, just prior to droplet extension, and at maximum extension. Mean ± 1 standard error, T = t test for normally distributed values; W = Wilcoxon test for values that were not normally distributed. (DOCX) [file pone.0196972.s007.docx]

S**5 Table. Comparison of axial line angles at droplet release and total loaded extension times between threads that were secured to sampler supports by carbon tape alone and those to which Elmer’s® glue was subsequently added.** Angles area compared at pre-extension, just prior to droplet extension, and at maximum extension. Mean ± 1 standard error, T = *t* test for normally distributed values; W = Wilcoxon test for values that were not normally distributed.

|  | *Argiope aurantia* N = 6 | | | *Neoscona crucifera* N = 6 | | | *Verrucosa arenata* N = 6 | | |
| --- | --- | --- | --- | --- | --- | --- | --- | --- | --- |
|  | **Tape + Glue** | **Tape** | ***P-*value** | **Tape + Glue** | **Tape** | ***P-*value** | **Tape + Glue** | **Tape** | ***P-*value** |
| **Pre-Extension** |  | | |  | | |  | | |
| 30% RH | 155 $\pm$ 4.7 | 150 $\pm$ 4.3 | T 0.5207 | -- | -- | -- | 144 $\pm2.9$ | 146 $\pm2.9$ | T 0.6073 |
| 50% RH | 150 $\pm$6.6 | 152 $\pm6.6$ | T 0.8511 | 144 $\pm7.8$ | 143 $\pm8.6$ | T 0.9310 | 129 $\pm6.0$ | 121 $\pm6.0$ | T 0.4039 |
| 70% RH | 161 $\pm4.0$ | 153 $\pm3.7$ | T 0.1730 | 148 $\pm5.2$ | 141 $\pm5.2$ | T 0.3642 | 134 $\pm6.6$ | 131 $\pm5.4$ | T 0.7460 |
| 90% RH | 161 $\pm2.3$ | 149 $\pm2.5$ | T **0.0035** | 136 $\pm5.0$ | 137 $\pm5.0$ | T 0.9449 | 134 $\pm6.9$ | 123 $\pm6.9$ | T 0.2594 |
| **50% Extension** |  | | |  | | |  | | |
| 30% RH | 143 $\pm7.0$ | 138 $\pm6.1$ | T 0.5804 | -- | -- | -- | 137 $\pm3.2$ | 140 $\pm3.2$ | T 0.5006 |
| 50% RH | 157 $\pm10.3$ | 158 $\pm9.4$ | T 0.9408 | 133 $\pm7.6$ | 133 $\pm8.4$ | T 1.0000 | 123 $\pm6.2$ | 115 $\pm5.3$ | W 0.2615 |
| 70% RH | 174.7 $\pm1.0$ | 168 $\pm3.9$ | W 0.1528 | 139 $\pm4.6$ | 132 $\pm4.6$ | T 0.2642 | 131 $\pm6.8$ | 129 $\pm5.5$ | T 0.8563 |
| 90% RH | 176 $\pm0.5$ | 173 $\pm0.5$ | W **0.0062** | 124 $\pm5.1$ | 126 $\pm5.2$ | W 0.9361 | 129 $\pm6.9$ | 121 $\pm6.9$ | T 0.4373 |
| **Maximum extension** |  | | |  | | |  | | |
| 30% RH | 141 $\pm10.0$ | 141 $\pm8.9$ | T 0.9645 | -- | -- | -- | 134 $\pm3.7$ | 133 $\pm3.7$ | T 0.8773 |
| 50% RH | 170 $\pm5.7$ | 165 $\pm7.0$ | W 0.6285 | 122 $\pm7.9$ | 125 $\pm8.6$ | T 0.7816 | 120 $\pm5.7$ | 110 $\pm5.7$ | T 0.2441 |
| 70% RH | 178 $\pm0.8$ | 174 $\pm2.6$ | W 0.4266 | 131 $\pm4.2$ | 123 $\pm4.2$ | T 0.2044 | 126 $\pm7.7$ | 125 $\pm6.3$ | T 0.9169 |
| 90% RH | 178 $\pm0.5$ | 177 $\pm0.6$ | W 0.2042 | 116 $\pm7.0$ | 118 $\pm7.0$ | W 0.8723 | 127 $\pm8.2$ | 122 $\pm8.2$ | T 0.6570 |
| **Loaded Time** |  | | |  |  |  |  | | |
| 30% RH | 27 $\pm2.5$ | 28 $\pm2.1$ | T 0.7757 | -- | -- | -- | 13 $\pm1.2$ | 14 $\pm1.2$ | T 0.6145 |
| 50% RH | 20 $\pm5$ | 23 $\pm5$.0 | T 0.6922 | 18.8 $\pm3.0$ | 16.6 $\pm3.3$ | T 0.6331 | 21 $\pm2.2$ | 24 $\pm2.2$ | T 0.3798 |
| 70% RH | 12 $\pm5.0$ | 17 $\pm4.1$ | T 0.4437 | 18 $\pm1.6$ | 20 $\pm1.6$ | T 0.2488 | 22 $\pm1.5$ | 22 $\pm1.2$ | T 0.8898 |
| 90% RH | 7 $\pm0.8$ | 13 $\pm2.4$ | T **0.0319** | 38 $\pm3.1$ | 35 $\pm3.1$ | T 0.4512 | 26 $\pm1.8$ | 26 $\pm1.8$ | T 0.8353 |
