# Supplementary material for: Elastic modulus and toughness of orb spider glycoprotein glue
Source: PLoS One. 2018 May 30;13(5):e0196972. doi: 10.1371/journal.pone.0196972 (PMC5976159; doi:10.1371/journal.pone.0196972)
Supplement: S6 Table — Mean ± 1 standard error. (DOCX) [file pone.0196972.s008.docx]

**S6 Table. *Argiope aurantia* axial line deflection, computed force on extended droplet, and droplet length from 25% - full extension.** Mean ± 1 standard error.

| *N* = 14 | | Axial Line Angle $∡$ | Glycoprotein Filament Force $\mu$N | Droplet Length $\mu$m |
| --- | --- | --- | --- | --- |
| **25%**  **Extension** | |  |  |  |
|  | 20% RH | 149 $\pm$ 3.3 | 9.4 $\pm$ 2.5 | 107 $\pm$ 17.8 |
|  | 37% RH | 156 $\pm$ 4.6 | 8.6 $\pm$ 3.8 | 374 $\pm$ 86.9 |
|  | 55% RH | 164 $\pm$ 3.8 | 3.4 $\pm$ 2.2 | 353 $\pm$ 69.7 |
|  | 72% RH | 162 $\pm$ 5.1 | 9.0 $\pm$ 7.0 | 535 $\pm$ 136.1 |
|  | 90% RH | 167 $\pm$ 4.3 | 5.2 $\pm$ 4.9 | 835 $\pm$ 144.1 |
| **50% Extension** | |  |  |  |
|  | 20% RH | 145 $\pm$ 3.8 | 12.3 $\pm$ 3.2 | 187 $\pm$ 28.7 |
|  | 37% RH | 156 $\pm$ 5.6 | 10.2 $\pm$ 5.0 | 667 $\pm$ 133.6 |
|  | 55% RH | 166 $\pm$ 4.2 | 3.4 $\pm$ 2.2 | 600 $\pm$ 93.1 |
|  | 72% RH | 168 $\pm$ 3.9 | 4.2 $\pm$ 3.1 | 827 $\pm$ 141.2 |
|  | 90% RH | 171 $\pm$ 4.3 | 4.6 $\pm$ 4.5 | 1132 $\pm$ 173.6 |
| **75% Extension** | |  |  |  |
|  | 20% RH | 142 $\pm$ 4.3 | 18.9 $\pm$ 5.6 | 298 $\pm$ 44.1 |
|  | 37% RH | 158 $\pm$ 5.7 | 10.6 $\pm$ 5.5 | 998 $\pm$ 162.4 |
|  | 55% RH | 171 $\pm$ 3.2 | 1.4 $\pm$ 0.9 | 804 $\pm$ 127.1 |
|  | 72% RH | 172 $\pm$ 2.2 | 0.6 $\pm$ 0.4 | 978 $\pm$ 159.4 |
|  | 90% RH | 177 $\pm$ 0.6 | 0.015 $\pm$0.009 | 1452 $\pm$ 203.4 |
| **Full Extension** | |  |  |  |
|  | 20% RH | 143 $\pm$ 4.7 | 19.7 $\pm$ 6.4 | 495 $\pm$ 68.5 |
|  | 37% RH | 161 $\pm$ 5.5 | 8.8 $\pm$ 5.2 | 1392 $\pm$ 176.3 |
|  | 55% RH | 171 $\pm$ 3.9 | 2.4 $\pm$ 1.6 | 1029 $\pm$ 156.4 |
|  | 72% RH | 177 $\pm$ 0.6 | 0.014 $\pm$ 0.007 | 1114 $\pm$ 183.3 |
|  | 90% RH | 178 $\pm$ 0.3 | 0.003 $\pm$ 0.001 | 1639 $\pm$ 253.7 |
