# Supplementary material for: Elastic modulus and toughness of orb spider glycoprotein glue
Source: PLoS One. 2018 May 30;13(5):e0196972. doi: 10.1371/journal.pone.0196972 (PMC5976159; doi:10.1371/journal.pone.0196972)
Supplement: S7 Table — Mean ± 1 standard error. (DOCX) [file pone.0196972.s009.docx]

**S7 Table. *Neoscona crucifera* axial line deflection, computed force on extended droplet, and droplet length from 25% - full extension.** Mean ± 1 standard error.

| Force/Extension Values – *Neoscona crucifera* | | | | |
| --- | --- | --- | --- | --- |
| *N* = 14 | | Axial Line Angle $∡$ | Glycoprotein Filament Force $\mu$N | Droplet Length $\mu$m |
| **25% Extension** | |  |  |  |
|  | 20% RH | -- | -- | -- |
|  | 37% RH | 156 $\pm$ 1.3 | 1.5 $\pm$ 0.2 | 37 $\pm$ 6.3 |
|  | 55% RH | 150 $\pm$ 2.4 | 3.2 $\pm$ 0.8 | 121 $\pm$ 19.3 |
|  | 72% RH | 144 $\pm$ 1.9 | 5.1 $\pm$ 0.8 | 336 $\pm$ 18.3 |
|  | 90% RH | 147 $\pm$ 3.9 | 5.2 $\pm$ 1.3 | 404 $\pm$ 48.2 |
| **50% Extension** | |  |  |  |
|  | 20% RH | -- | -- | -- |
|  | 37% RH | 152 $\pm$ 1.3 | 1.9 $\pm$ 0.3 | 78 $\pm$ 10.8 |
|  | 55% RH | 146 $\pm$ 3.0 | 4.3 $\pm$ 1.1 | 191 $\pm$ 27.0 |
|  | 72% RH | 139 $\pm$ 2.2 | 6.7 $\pm$ 1.1 | 528 $\pm$ 40.8 |
|  | 90% RH | 145 $\pm$ 5.8 | 7.2 $\pm$ 1.9 | 709 $\pm$ 94.5 |
| **75% Extension** | |  |  |  |
|  | 20% RH | -- | -- | -- |
|  | 37% RH | 150 $\pm$ 1.2 | 2.8 $\pm$ 0.3 | 110 $\pm$ 13.4 |
|  | 55% RH | 143 $\pm$ 3.6 | 7.0 $\pm$ 2.0 | 280 $\pm$ 38.0 |
|  | 72% RH | 135 $\pm$ 2.3 | 10.0 $\pm$ 1.4 | 756 $\pm$ 70.0 |
|  | 90% RH | 144 $\pm$ 7.0 | 10.9 $\pm$ 3.1 | 1070 $\pm$ 138.3 |
| **Full Extension** | |  |  |  |
|  | 20% RH | -- | -- | -- |
|  | 37% RH | 148 $\pm$ 1.5 | 3.5 $\pm$ 0.5 | 170 $\pm$ 19.5 |
|  | 55% RH | 142 $\pm$ 3.8 | 7.7 $\pm$ 2.3 | 444 $\pm$ 66.4 |
|  | 72% RH | 134 $\pm$ 2.6 | 11.1 $\pm$ 1.8 | 1062 $\pm$ 101.0 |
|  | 90% RH | 146 $\pm$ 7.7 | 11.9 $\pm$ 3.7 | 1500 $\pm$ 191.5 |
|  |  |  |  |  |
