# Supplementary material for: Elastic modulus and toughness of orb spider glycoprotein glue
Source: PLoS One. 2018 May 30;13(5):e0196972. doi: 10.1371/journal.pone.0196972 (PMC5976159; doi:10.1371/journal.pone.0196972)
Supplement: S8 Table — Mean ± 1 standard error. (DOCX) [file pone.0196972.s010.docx]

**S8 Table. *Verrucosa arenata* axial line deflection, computed force on extended droplet, and droplet length from 25% - full extension.** Mean ± 1 standard error.

| *N* = 11-12 | | Axial Line Angle $∡$ | Glycoprotein Filament Force $\mu$N | Droplet Length $\mu$m |
| --- | --- | --- | --- | --- |
| **25% Extension** | |  |  |  |
|  | 20% RH | 148 $\pm$ 3.6 | 10.9 $\pm$ 4.4 | 9 $\pm$ 3.7 |
|  | 37% RH | 133 $\pm$ 2.6 | 27.7 $\pm$ 3.8 | 36 $\pm$ 5.4 |
|  | 55% RH | 126 $\pm$ 3.2 | 43.6 $\pm$ 7.7 | 72 $\pm$ 174.0 |
|  | 72% RH | 127 $\pm$ 3.7 | 43.0 $\pm$ 6.9 | 215 $\pm$ 39.2 |
|  | 90% RH | 123 $\pm$ 6.0 | 64.8 $\pm$ 16.5 | 428 $\pm$ 50.7 |
| **50% Extension** | |  |  |  |
|  | 20% RH | 147 $\pm$ 3.8 | 11.9 $\pm$ 4.6 | 11 $\pm$ 5.2 |
|  | 37% RH | 127 $\pm$ 2.8 | 34.9 $\pm$ 4.4 | 77 $\pm$ 13.9 |
|  | 55% RH | 121 $\pm$ 3.5 | 53.3 $\pm$ 9.8 | 150 $\pm$ 28.6 |
|  | 72% RH | 120 $\pm$ 3.8 | 55.5 $\pm$ 8.7 | 378 $\pm$ 67.7 |
|  | 90% RH | 119 $\pm$ 6.9 | 77.3 $\pm$ 19.8 | 762 $\pm$ 95.5 |
| **75% Extension** | |  |  |  |
|  | 20% RH | 146 $\pm$ 4.0 | 13.7 $\pm$ 5.0 | 14 $\pm$ 6.6 |
|  | 37% RH | 122 $\pm$ 3.0 | 52.1 $\pm$ 6.6 | 121 $\pm$ 19.1 |
|  | 55% RH | 117 $\pm$ 3.6 | 72.2 $\pm$ 13.5 | 251 $\pm$ 45.2 |
|  | 72% RH | 114 $\pm$ 4.4 | 84.6 $\pm$ 14.8 | 579 $\pm$ 106.8 |
|  | 90% RH | 120 $\pm$ 8.6 | 94 $\pm$ 24.4 | 1211 $\pm$ 141.7 |
| **Full Extension** | |  |  |  |
|  | 20% RH | 145 $\pm$ 4.3 | 15.7 $\pm$ 5.8 | 19 $\pm$ 8.2 |
|  | 37% RH | 118 $\pm$ 3.3 | 66.8 $\pm$ 9.3 | 194 $\pm$ 23.1 |
|  | 55% RH | 115 $\pm$ 3.7 | 79.8 $\pm$ 15.0 | 414 $\pm$ 68.1 |
|  | 72% RH | 113 $\pm$ 4.2 | 86.8 $\pm$ 14.2 | 900 $\pm$ 150.6 |
|  | 90% RH | 123 $\pm$ 9.1 | 89.7 $\pm$ 25.0 | 1749 $\pm$ 193.1 |
